# Persistent lumbar radicular and low back pain; impact of genetic variability versus emotional distress

**DOI:** 10.1186/s13104-019-4592-6

**Published:** 2019-08-28

**Authors:** Siri Bjorland, Johannes Gjerstad, Elina Schistad, David M. Swanson, Cecilie Røe

**Affiliations:** 1 Faculty of Medicine, University of Oslo, Postboks 1078 Blindern, 0316 Oslo, Norway; 2 Department of Physical Medicine and Rehabilitation, Oslo University Hospital Ullevål, Postboks 4956 Nydalen, 0424 Oslo, Norway; 3 National Institute of Occupational Health, Gydas vei 8, 0363 Oslo, Norway; 4 Department of Molecular Bioscience, University of Oslo, Postboks 1066 Blindern, 0316 Oslo, Norway; 5 Department of Biostatistics, University of Oslo, Postboks 1078 Blindern, 0316 Oslo, Norway

**Keywords:** Low back pain, Lumbar radicular pain, Emotional distress, Genetic variability

## Abstract

**Objective:**

Earlier studies documenting the effect of candidate genes on recovery have seldom taken into consideration the impact of emotional distress. Thus, we aimed to assess the modifying effect of emotional distress on genetic variability as a predictor for pain recovery in lumbar radicular (LRP) and low back pain (LBP).

**Results:**

The study population comprised 201 patients and mean age was 41.7 years. The significant association between MMP9 rs17576 (B = 0.71, 95% CI 0.18 to 1.24, p = 0.009) and pain recovery remained statistically significant after adjusting for pain intensity at baseline, age, gender, smoking, body mass index, pain localization and emotional distress (B = 0.68, 95% CI 0.18 to 1.18, p = 0.008). In contrast, the association between OPRM1 (B = − 0.85, 95% CI − 1.66 to − 0.05, p = 0.038) and pain recovery was abolished in the multivariate analysis (B = − 0.72, 95% CI − 1.46 to 0.02, p = 0.058). Hence, MMP9 rs17576 and emotional distress independently seem to predict persistent back pain. The predictive effect of OPRM1 rs179971 with regard to the same outcome is probably dependent on other factors including emotional processing.

*Trial registration* The Regional Committee for Medical Research and Ethics reference number 2014/1754

## Introduction

Persistent low back pain (LBP) has a point prevalence of 30% and creates a substantial personal and financial public burden globally [1–6]. Lumbar radicular pain (LRP), also referred to as sciatica, accounts for 5–10% of these LBP conditions. Although most back disorders are benign, many patients have a slow recovery [7–9]. In patients with pain, symptoms often tend to be associated with each other, and back pain may be only one of several possible symptoms of general frailty [10]. Psychological factors are of major importance for poor recovery and transition into chronic pain [11, 12]. Actually, many patients focus too much on their bodily symptoms such as dizziness, fatigue or insomnia. Such symptoms in absence of specific diseases in combination with bad mood are often termed emotional distress [11, 12]. Longitudinal studies suggest that major depression increases the risk of developing future chronic pain [13, 14], and a recent study showed that psychological factors such as emotional exhaustion, mental distress, having little surplus, feeling depressed may propagate the spread of pain [15].

Earlier data suggest that the heritability of back pain range from 30 to 45% [16]. Genetic variability, which is important for degenerative changes, inflammation or pain perception may play a role in both LBP and LRP conditions [17–21]. Genetic variants in genes encoding proteins such as vitamin D receptor (VDR), collagens (COL) and matrix metalloproteinases (MMPs) may affect degeneration of the intervertebral discs [18, 22]. In addition, the relationship between pain and MMP9, which is also involved in immunomodulation [23], neuromodulation [24], and activation of the epiregulin—PI3K/AKT/mTOR pathway [25], may affect pain sensitivity. Moreover, previous studies indicate that genetic polymorphisms related to inflammation in genes encoding interleukin 1 (IL-1α), interleukin-1 receptor antagonist (IL-1RN) and interleukin-6 (IL-6) may promote persistent LRP [26–30].

Some of the same genetic polymorphisms may also be associated with supra-spinal neuronal activity including stress-induced depression [31]. Moreover, genetic variability related to opioid, dopaminergic, adrenergic and serotonergic signaling affect perceptual modulation and nociceptive processing [32–34]. For example, genetic variability related to the enzyme catechol-*O*-methyltransferase (COMT) affects cortical pain processing and the risk of long-lasting pain conditions [35–37]. In addition, several earlier studies have demonstrated a link between genetic variability in the gene encoding opioid receptor mu 1 (OPRM1) and pain recovery in LRP patients [38–40]. Finally, the OPRM1 variant may be associated with attenuated hypothalamic–pituitary–adrenal (HPA) axis responses to stress [41]. The OPRM1 variant is also associated with personality traits [42] and subjective health complaints [39].

Taken together, these observations and similar data from other studies suggest that genetic factors linked to pain recovery, may be associated with peripheral inflammation—but also neuronal activity in brain regions associated with emotions. In addition, recent data from our group have documented a robust association between the MMP9 rs17576 as well as OPRM1 rs1799971 variants and pain recovery [40]. Hence, the aim of the present study was to examine the modifying effect of emotional distress on the association between genetic variability and pain recovery.

## Main text

### Study design and study population

The dataset comprises two ongoing prospective cohorts, which are merged after inclusion but before 5-year follow-up. The methods are previous thoroughly described in our publication in PAIN; “Genetic predictors of recovery in low back and lumbar radicular pain” [40]. This study comprises a subsample of patients from this cohort. This subsample emerges from the Oslo University Hospital (OUH) Ullevaal due to assessment of emotional distress only in this hospital. In total, 275 patients with lumbar radicular pain (LRP) or low back pain (LBP) were recruited between 2007 and 2009. The inclusion criteria in both cohorts was age between 18 and 60 years, and exclusion criteria were specific spinal pathology, generalized musculoskeletal pain, inflammatory rheumatic diseases, diabetic polyneuropathy or serious diseases.

The study was conducted in accordance with the Helsinki Declaration. The Regional Committee for Medical Research Ethics (reference number 2014/1754) and the Norwegian Social Science Data Service approved the study protocol and all participants gave their written informed consent at baseline and at 5-year follow-up.

### Outcome measures

Emotional distress was assessed using a short version of Hopkins Symptoms Check List (HSCL-10), 4-point scale, where 1 = no complains, 2 = some complaints, 3 = moderate complaints and 4 = many complaints. The scores summarize and divide on the number of answered questions, with a mean score > 1.85 being compatible with emotional distress symptoms present [43].

Pain intensity was recorded using the visual analogue scale (VAS) with anchor values from 0 (no pain) to 10 (worst possible pain) at rest during the last week at baseline and at 5-year follow-up. A cut off ≥ 3.5 is defined as moderate pain [44].

### Genotyping

In patients with LRP, genomic DNA was collected at baseline and extracted from whole blood cells using a FlexiGene DNA isolation kit (Qiagen), whereas in patients with LBP genomic DNA was collected at 5-year follow up and extracted from saliva using an Oragene DNA sample collection kit (DNA Genotech Inc., California, USA) according to the manufacturer’s instructions. SNP genotyping was carried out using predesigned TaqMan SNP genotyping assays (Applied Biosystems). Genotypes were determined using the SDS 2.2 software (Applied Biosystems). Phase v.2.1.1 was used to define the COMT haplotypes. Approximately 10% of the samples were re-genotyped and the concordance rate was 100%.

### Genetic variants and statistical analysis

Descriptive statistics, t-test and Chi-square test were applied to describe the patients at baseline and 5-year follow-up and evaluate differences between LBP and LRP. Univariate linear regression analysis was performed to estimate the correlation between the eight genetic variants (VDR, COL11, MMP1, MMP9, IL-1α, IL-1RN, OPRM1 and COMT) and pain (VAS) at 5-year follow-up. Further on, we adjusted for emotional distress (HSCL-10) at baseline in the analysis to evaluate the impact of the psychosocial factor. Univariate linear regression analysis also performed to estimate the correlation between emotional distress and pain (VAS) at 5-year follow-up. Significant genetic variants p level < 0.05 were included in multivariable linear regression models where we adjusted for pain intensity at baseline, age, gender, smoking status (yes or no), body mass index, pain localization (LRP or LBP) and emotional distress. All models were checked for collinearity (no collinearity revealed). All statistical analyses were performed using the SPSS (version 22) statistical package. A p-value < 0.05 was defined as being statistically significant.

## Results

The study population comprised 201 patients, including 92 (46%) females and 109 males (54%) with ages of 18 to 59 (mean 41.7 ± 9.6). An active smoking habit was reported in 34% of the patients. At baseline, patients in both cohorts reported moderate pain intensity (mean VAS > 3.5) with significantly higher pain in LBP compared to LRP (p = 0.012). Regarding emotional distress, the LBP patients reported significantly more complaints (mean HSCL-10 > 1.85) than the LRP patients (mean HSCL-10 < 1.85) (p = 0.001). At 5-year follow up, the LBP patients reported significantly more pain (mean VAS > 3.5) than the LRP patients (mean VAS < 3.5) (p < 0.001). Improvement of emotional distress occurred in both the LBP (mean HSCL-10 < 1.85) and the LRP groups (mean HSCl-10 < 1.85), but a significant difference in emotional distress was seen (p = 0.035). Detailed characteristics of the study population are shown in Table 1.

**Table 1:** Characteristics of study population

	LBP n = 106	LRP n = 95	p value
Age			
Baseline	41.4 (9.2)	41.9 (10.0)	n.s^a^
Women			
Baseline	39.6 (42)	52.6 (50)	n.s^b^
Smoke			
Baseline	33.0 (35)	34.7 (33)	n.s^b^
5-year follow up	31 (33)	26 (25)	n.s^b^
Pain (VAS) rest			
Baseline	4.7 (2.3)	3.8 (2.5)	0.012^a^
5-year follow up	3.7 (2.9)	2.3 (2.2)	< 0.001^a^
Pain (VAS) activity			
Baseline	6.3 (2.2)	5.7 (2.7)	n.s^a^
5-year follow up	4.7 (3.0)	3.2 (2.8)	< 0.001^a^
Emotional distress (HSCL-10)			
Baseline	2.1 (0.6)	1.8 (0.5)	0.001^a^
5-year follow up	1.7 (O.6)	1.5 (0.6)	0.035^a^

Continual data*: mean* (*SD*). Categorical data: *Percent* (*n*)

*n.s* not significant

^a^Unpaired Student’s test

^b^Pearson Chi square test

In univariate linear regression analysis, emotional distress showed a highly significant association with pain intensity at 5-year follow-up (p < 0.001 and R^2^ = 0.094). Regarding the genetic variants, the univariate linear regression analysis showed that the associations between MMP9 rs17576 as well as OPRM1 rs1799971 and pain at 5 years remained statistically significant also in this subsample of patients (p = 0.009 and R^2^ = 0.034, p = 0.038 and R^2^ = 0.022). In the multivariable analysis adjusting for potential confounding effects of baseline pain, pain location (LRP or LBP), age, gender, smoking (yes or no), BMI and emotional distress, only MMP9 rs17576, not OPRM1 rs1799971, remained significant (p = 0.008) (Table 2a, b). In addition, pain location (LRP or LBP) showed a significant association with pain at 5 years in the multivariate model (p = 0.027).

**Table 2:** Multivariable linear regression with pain intensity at rest 5 years as dependent variable

	Unstandardized coefficients		Confidence interval		Sig.
	B	SE	Lower	Upper	
a)					
MMP9	0.68	0.25	0.18	1.18	0.008
Age	0.04	0.02	− 0.00	0.07	0.053
Gender	− 0.00	0.35	− 0.70	0.698	1.000
Smoking	0.25	0.37	− 0.48	0.97	0.856
BMI	0.01	0.03	− 0.05	0.07	0.856
Pain (VAS) rest baseline	0.34	0.08	0.19	0.49	< 0.001
Pain location (LRP or LBP)	0.79	0.36	0.09	1.49	0.027
Emotional distress (HSCL-10) baseline	0.74	0.32	0.10	1.38	0.023
R^2^					0.26
Adjusted R^2^					0.23
b)					
OPRM1	− 0.72	0.38	− 1.46	0.02	0.058
Age	0.03	0.02	− 0.01	0.07	0.089
Gender	− 0.19	0.35	− 0.89	0.51	0.586
Smoking	0.33	0.37	− 0.40	1.06	0.367
BMI	0.01	0.03	− 0.05	0.07	0.742
Pain (VAS) rest baseline	0.34	0.08	0.19	0.49	< 0.001
Pain location (LRP or LBP)	0.88	0.36	0.18	1.59	0.014
Emotional distress (HSCL-10) baseline	0.64	0.33	− 0.01	1.28	0.052
R^2^					0.25
Adjusted R^2^					0.21

Multivariable linear regression with pain intensity (VAS) at rest at 5 years as dependent variable. a) MMP9 as predictor adjusted for demographic and clinical confounders and controlled for emotional distress. b) OPRM1 as predictor adjusted for demographic and clinical confounders and controlled for emotional distress

## Discussion

Recent data from our group have demonstrated that both MMP9 rs17576 and OPRM1 rs1799971 may affect 5-year recovery in patients with LRP and LBP [40]. In the present study, we have extended these findings and shown that MMP9 rs17576 remains a significant predictor also when controlling for emotional distress. Thus, the MMP9 rs17576 did not affect pain recovery through emotional distress. In contrast, our data suggested that the OPRM1 rs179971—pain relationship may be more complex—and be dependent on emotional processes. This result may be related to OPRM1 having a role in a common supra-spinal neural network processing affective component of pain. Earlier data have also demonstrated that brain regions particular related to the somatosensory component of pain processing may be moderated by genetic variations in the gene encoding OPRM1 [45].

The MMP9 genotype explained 2.2% of the variation of pain at 5 years. Emotional distress on the other hand explained 9.4% of the variation of pain at 5 years. These findings support the previous observation that psychological distress is a major predictor for persistent back pain [46]. The LBP patients reported significantly more emotional complaints than the LRP patients both at baseline and 5-year follow-up. However, the data suggested that poorer pain recovery in LBP cannot be explained by the emotional factor alone. Psychological factors at baseline are shown to correlate with persistent LBP [47]. However, earlier data suggest that emotional distress may not be a strong predictor for persistence of low back disability in persons having their first episode of LBP [48]. Nevertheless, to prevent persistent back disability, emotional distress should definitely be considered and treated [48].

In contrast to earlier studies with shorter follow-up [49], the LRP patients showed significantly better pain recovery than the LBP patients, even when controlling for emotional distress. This result suggests that the pain mechanism may change over time. Another explanation could be that LRP patients and LBP patients referred to specialized healthcare are different from the start [50]. Complex disorders such as LRP and LBP are multifactorial pain conditions [51–53] and whether or not application of results obtained from genetic studies really are ready for clinical use is controversial [54]. However, persistent back pain also includes a biological aetiology [55]. Still, synthesis of clinical and biological research should be important for a more rational management of persistent back pain in the future [56].

In conclusion, the present study showed that MMP9 rs17576 and emotional distress independently predict persistent back pain. OPRM1 rs179971 predicts the same outcome, but for this genetic variant, the effect may be dependent on emotional processing.

## Limitations

Being based on a mix of LRP and LBP patients, the present study has its limitations. We would, however, argue that the subsample of patients included in the present study is representative for the cohort. Still, the mix of LBP and LRP may reduce statistical power. Also, although, the short version of Hopkins Symptoms Checklist (HSCL-10) is a valid instrument [57], it does not distinguish between anxiety, depression, somatization or other psychological symptoms. Hence, HSCL-10 may not specify the type of the mental problem in our patients. Finally, the use of the candidate approach will not discover all genetic factors influencing pain.

## Data Availability

The dataset analyzed during the current study are not publicly available due to the data handling rules of health region south east, Norway, but are available from the corresponding author on reasonable request.

## Abbreviations

COMT: catechol-*O*-methyltransferase
COL: collagen
HSCL-10: Hopkins Symptoms Check List
IL 1 α: interleukin 1 alfa
LBP: low back pain
LRP: lumbar radicular pain
MMP: matrix metalloproteinase
OPRM1: opioid receptor mu 1
SNP: single nucleotide polymorphism
VAS: visual analogue scale
VDR: vitamin D receptor
